# Exploring Potential of Gellan Gum for Enhanced Oil Recovery

**DOI:** 10.3390/gels9110858

**Published:** 2023-10-29

**Authors:** Iskander Gussenov, Ramza Zh. Berzhanova, Togzhan D. Mukasheva, Gulnur S. Tatykhanova, Bakyt A. Imanbayev, Marat S. Sagyndikov, Sarkyt E. Kudaibergenov

**Affiliations:** 1 Institute of Polymer Materials and Technology, microdistrict “Atyrau 1”, 3/1, Almaty 050019, Kazakhstan; gulnur-ts81@yandex.ru; 2 Petroleum Engineering Department, Satbayev University, Satbayev str. 22a, Almaty 050043, Kazakhstan; 3 Faculty of Biology and Biotechnology, al-Farabi Kazakh National University, 71 al-Farabi Ave., Almaty 050040, Kazakhstan; ramza05@mail.ru (R.Z.B.);; 4 KMG Engineering LLP, 35 mkr, plot 6/1, Aktau R00P0D6, Kazakhstan; b.imanbayev@kmge.kz (B.A.I.);

**Keywords:** gellan, polysaccharides, hydrolyzed polyacrylamide, enhanced oil recovery, plugging, injection well, production well

## Abstract

Extensive laboratory and field tests have shown that the gelation response of gellan gum to saline water makes it a promising candidate for enhanced oil recovery (EOR). The objective of this mini-review is to evaluate the applicability of gellan gum in EOR and compare its efficiency to other precursors, in particular, hydrolyzed polyacrylamide (HPAM). At first, the “sol-gel” phase transitions of gellan gum in aqueous-salt solutions containing mono- and divalent cations are considered. Then the rheological and mechanical properties of gellan in diluted aqueous solutions and gel state are outlined. The main attention is paid to laboratory core flooding and field pilot tests. The plugging behavior of gellan in laboratory conditions due to “sol-gel” phase transition is discussed in the context of conformance control and water shut-off. Due to its higher strength, gellan gum gel provided ~6 times greater resistance to the flow of brine in a 1 mm-width fracture compared to HPAM gel. The field trials carried out in the injection and production wells of the Kumkol oilfield, situated in Kazakhstan, demonstrated that over 6 and 11 months, there was an incremental oil recovery of 3790 and 5890 tons, respectively. To put it into perspective, using 1 kg of dry gellan resulted in the incremental production of 3.52 m^3^ (or 22 bbls) of oil. The treatment of the production well with 1 wt.% gellan solution resulted in a considerable decrease in the water cut up to 10–20% without affecting the oil flow rate. The advantages and disadvantages of gellan compared to HPAM are analyzed together with the economic feasibility of gellan over HPAM. The potential for establishing gellan production in Kazakhstan is emphasized. It is anticipated that gellan gum, manufactured through fermentation using glucose–fructose syrup from Zharkent and Burunday corn starch plants, could be expanded in the future for applications in both the food industry and oil recovery.

## 1. Introduction

Conventional water flooding often falls short of achieving sustainable oil recovery due to challenges such as viscous fingering and water breakthrough via high-permeability channels. Addressing these issues necessitates the application of synthetic or biopolymers. These agents enable an increase in the viscosity of the oil recovery drive fluid or the plugging of high-permeability channels, ultimately enhancing sweep efficiency [1,2,3].

The utilization of polysaccharides in oil recovery has been thoroughly documented and was recently discussed by the authors [1,2]. The primary focus was on xanthan [3,4,5,6,7,8,9,10], guar gum [11,12], scleroglucan [5,13], welan [14], carboxymethyl- and hydroxyethylcellulose [15,16], starch [17], diutan, pullulan [18,19], carrageenan [20,21], and, to a lesser degree, gellan [22,23]. A comparative study of polysaccharides (xanthan gum, diutan gum, and scleroglucan) and synthetic polymers, mainly partially hydrolyzed poly(acrylamide) (HPAM), with respect to EOR was carried out by authors [24]. The study revealed that polysaccharides exhibited lower sensitivity to salinity when compared to HPAM. The findings demonstrated that both diutan gum and scleroglucan displayed significantly superior salt tolerance, long-lasting thermal stability, and a higher efficiency in oil recovery compared to HPAM and xanthan gum. For example, in high salinity and temperature conditions, the oil recovery from a sand pack by diutan gum was equal to 20.9%, whereas xanthan and HPAM recovered only 9.3% and 5.4% of oil, respectively [9]. Due to the synergistic effect, the mixtures of xanthan–gellan and welan–gellan possess enhanced rheological and gel-forming properties in the presence of mono-, di-, and trivalent cations (Na^+^, K^+^, Ca^2+^, and Al^3+^) and can be used for oil recovery [25]. Most polysaccharides belong to anionic polyelectrolytes due to the presence of ionizable carboxylic or sulfonic groups in macromolecular chains and are sensitive to temperature, pH, and salinity. The molecular weights of welan, gellan, diutan, xanthan, and scleroglucan are arranged between 6 × 10^5^–5 × 10^6^ Dalton [24,25]. In reservoir conditions, the main requirements for polysaccharides are thermal-, salt-, and biostability. Polysaccharides are suitable candidates for EOR in high temperatures and high salinity. The thermal and duration stability of some polysaccharides and HPAM is compared in Table 1.

Reservoir conditions (temperature, salinity, rock surface charges, and the characteristics of the crude oil) are crucial considerations for polymer-augmented EOR. In comparison to synthetic polymers conventionally employed for EOR, biopolymers offer several advantages: (1) thermal stability (up to 150 °C in some cases), (2) mechanical resilience, (3) high salinity tolerance, (4) stability across a wide range of pHs, and (5) environmental safety. While there have been numerous studies on gellan and its modified derivatives, in our opinion, there has been insufficient emphasis on systematically testing and applying gellan for EOR. This review, drawing from existing literature and our own findings, aims to address this gap.

## 2. Conformational and Phase Behaviors of Gellan Gum in Aqueous-Salt Solutions and Oilfield Saline Water

One of the advantages of gellan over the rest of the polysaccharides in EOR is the quick gelation in saline water. In reservoir conditions, this is beneficial from several points of view: (1) preparation of the desired concentration of polymer in low-salinity water (1 g∙L^−1^) in the range of 0.1–1.0 wt.% or higher; (2) easy pumping of gellan solution into the well; (3) easy regulation of the gellan concentration upon injection; (4) fast gelation of the gellan upon contact with the saline reservoir water; (5) the absence of necessity to inject crosslinking agents, for example, Al^3+^, Cr^3+^, Fe^3+^, and Zr^4+^, as in case of HPAM; (6) ecological safety and friendless of gellan due to biodegradation.

The structure, physico-chemical properties, conformational and phase transition of gellan gum triggered by the change in temperature, the addition of ions, and the pH medium were comprehensively reviewed by Morris, Nishinari, and Rinaudo [33]. Commercially fermented gellan gum contains residual mono- and divalent cations from nutrient salts used in bacterial growth and post-fermentation processing (see Table 2) [34].

Use of the tetramethylammonium (TMA) salt N(CH_3_)_4_^+^Cl^−^ avoids gel formation, as reported by authors [34], because gelation of gellan with TMA cations occurs only at very high concentrations, as is distinct from K^+^, Na^+^, Ca^2+^, and Mg^2+^. In this connection, the molecular characteristics of gellan gum were measured in 0.025 mol∙L^−1^ TMA at 25 °C, which is supposed to prevent the aggregation phenomena. As a result, the Mark–Kuhn–Houwink equation: [η] = 7.48∙10^−3^∙M_w_^0.91^ was suggested by authors [35] to calculate the viscosity–average molecular weights (M_η_) from the viscosimetric measurements.

Gellan gum is classified as a thermoresponsive polymer, demonstrating a reversible conformational change at approximately 30 °C. This transition takes it from a disordered state (single coil) at higher temperatures to an organized state (double helix) at lower temperatures [36]. The cooling enhances the shifting of gellan molecules from a disordered coil conformation to an organized helical structure. The influence of the molar mass of sodium-type gellan on the coil–helix transition was investigated in an aqueous solution containing 25 mmol of NaCl [37]. In fact, the transition from coil to double helix for polymers with an M_w_ equal to 120 and 32 kDa occurs at around 30 °C. Of note is that the transition from coil to double helix is expedited with an increase in M_w_. The minimum molar mass required for helix formation falls within the range of 17–32 kDa. The impact of various cations on the conformational properties of gellan gum in a water-based solution was investigated. This was achieved by examining highly purified forms of gellan with lithium, sodium, and potassium and assessing parameters such as osmotic pressure, intrinsic viscosity, and circular dichroism [38]. In an aqueous solution without added salts, the transition temperatures for Li-, Na-, and K-gellans are the same, while the coil–double helix conformational transition rises as the polymer concentration increases. This trend was observed regardless of the specific type of cation present. Increasing the salt concentrations enhances the formation of double helix conformation due to the shielding effects of the countercations in the following sequence: K-gellan > Li-gellan > Na-gellan. This is probably due to the structure disordering ability of K^+^ with respect to water molecules in contrast to the structure ordering tendency of Na^+^ and Li^+^. Hydrogen bonds between hydroxyl groups of gellan also play an important role in enhancing the formation of a double helix [39].

A model for gellan gelation in the presence of mono- and divalent cations was proposed [40]. The coil–helix (T_CH_) and sol-gel (T_SG_) transition temperatures in gellan gum solutions were detected by both thermal scanning rheology and differential scanning calorimetry (DSC). The gelation efficiency of gellan gum depends on the nature of the added salt and is followed by the Hofmeister series Cs^+^ > K^+^ > Na^+^ > Li^+^.

The gelation mechanism of gellan gum in the presence of divalent cations is substantially different from monovalent cations. At T > T_CH_, cooling leads to immediate interaction of gellan gum segments with divalent cations to form the specific ordered structures. Further addition of divalent cations stabilizes these ordered structures, and the system becomes extremely thermostable. The specific cation–polyanion interaction leads to the formation of an elastic gel.

**Figure 1:**
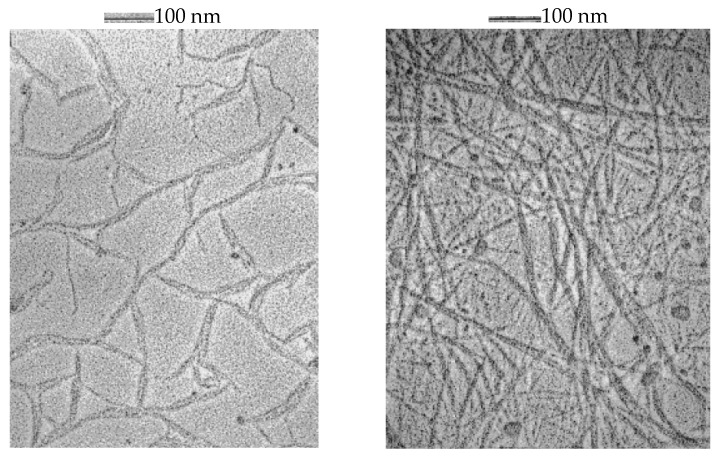
Gellan morphology at 10 mg·mL^−1^ in the presence of 25 mM Ca^2+^ (**left**) and 50 mM K^+^ (**right**). Reprinted from Ref. [41].

The morphology of gellan in the absence and presence of K^+^ and Ca^2+^ was directly visualized via transmission electron microscopy (TEM) (Figure 1) [41].

TEM images suggest that without salt, the most thermodynamically stable conformation of gellan in an aqueous solution is the double helix and double-helical duplexes. Both K^+^ and Ca^2+^ induce gelation-bridging double helices. The strength of the gel depends on the strength of the junction zones and is determined by the number of polymers involved in the gelation process.

To image the gel formation of gellan gum, the atomic force microscope (AFM) method was used [42]. The gellan solution prepared in n-butanol with a concentration of 10 mg·mL^−1^ after drying and deposition onto freshly cleaved mica revealed the formation of continuous branched fibrous networks in the form of elongated and/or branched aggregates. In the presence of gel-promoting cations, a single fibrous network was formed. Due to the binding of gellan helices by ions, the side-by-side junction zones were formed within the fibers.

It is commonly accepted that the gelation of gellan in the presence of mono- and divalent alkaline and alkaline earth cations is due to the formation of a double helix structure [33]. Gel formation upon the addition of cations follows the order: Cs^+^ > Rb^+^ > K^+^ > Na^+^ > Li^+^ in accordance with increasing the ionic radius of the cation species. The effectiveness of cations to enhance gelation decreases in the order Ca^2+^ > Mg^2+^ > K^+^ > Na^+^. Divalent cations bind the gellan helices with the effectiveness of Ca^2+^ >> Mg^2+^ [43]. The Ca^2+^ induces a denser filling structure of gellan than that of K^+^ [44].

**Figure 2:**
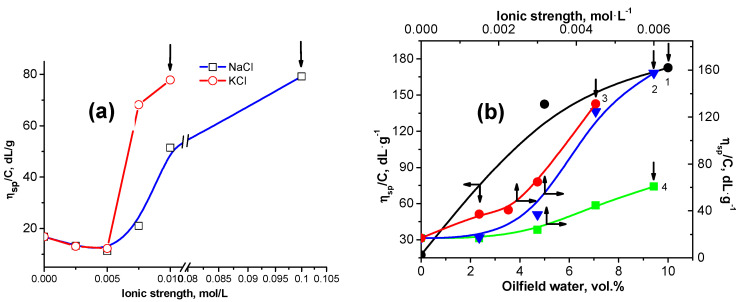
(**a**) Changing of the reduced viscosity of 0.2 wt.% gellan upon addition of NaCl and KCl at 25 °C. Arrows show the start of the gelation process. (**b**) Changing of the reduced viscosity of 0.2 wt.% gellan upon addition of oilfield water with a salinity of 73 g∙L^−1^ (1), CaCl_2_ (2), BaCl_2_ (3), MgCl_2_ (4) at 25 °C. Arrows show the start of the gelation process [45,46,47,48].

The reduced viscosity of 0.2 wt.% gellan upon the addition of NaCl, KCl, MgCl_2_, CaCl_2_, BaCl_2_ is shown in Figure 2 [45,46,47,48].

The effectiveness of salts to enhance gellan gelation changes in the following order: BaCl_2_ > CaCl_2_ ≈ MgCl_2_ > KCl > NaCl [45,46], which is in good agreement with the results of the authors [33]. The critical concentration of salts (C_crit_) leading to the sol-gel transition of 0.2 wt.% gellan solution is summarized in Table 3 [45,46].

**Figure 3:**
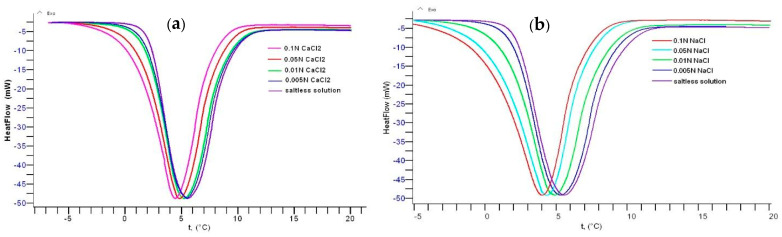
DSC curves of 0.5 wt.% gellan in the presence of various concentrations of CaCl_2_ (**a**) and NaCl (**b**) at a heating rate of 0.5 °C∙min^−1^. Ref. [45].

The phase transition temperature of gellan in aqueous-salt solutions containing various concentrations of NaCl and CaCl_2_ was evaluated with differential scanning calorimetry (DSC) measurements [46] (Figure 3). In the presence of 0.1 mol∙L^−1^ CaCl_2_ and NaCl, the endothermic peak of pristine gellan at 5.5 °C gradually shifts to 4.4 °C (∆T = 1.1 °C) and 4.0 °C (∆T = 1.5 °C), respectively. The difference in the endothermic behavior of gellan solution in the presence of CaCl_2_ and NaCl can be explained with the dissociation of “bridged” and “shielded” macromolecular chains by bivalent and monovalent cations [49].

Table 4 summarizes the specific heat capacity (∆C) of 0.5 wt.% gellan solution in the presence of various salts [39]. Increasing the ∆C at the interval of a salt concentration of 0.005–0.01 mol∙L^−1^ in the order: MgCl_2_ > CaCl_2_ > KCl > NaCl coincides well with the effectiveness of salts to enhance gellan gelation. However, this sequence is not performed at salt concentrations of 0.05 and 0.1 mol∙L^−1^.

**Figure 4:**
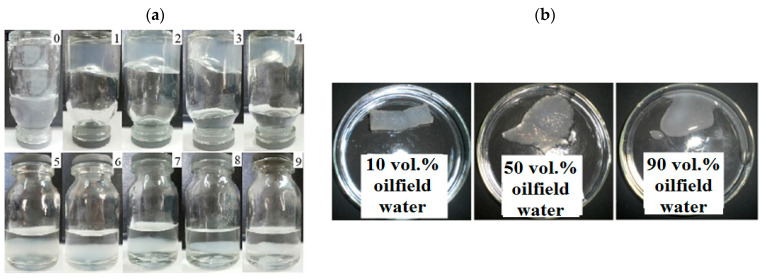
(**a**). Sol–gel transition (1–4) and liquid and dense solid phases formation of gellan (5–9) in the presence of 0 (0), 10 (1), 20 (2), 30 (3), 40 (4), 50 (5), 60 (6), 70 (7), 80 (8), and 90 vol.% oilfield water (9) with salinity of 73 g∙L^−1^. V_total_ = 5 mL, C_total_ = 0.5 wt.% [46]. (**b**). Transformation of gellan gel into the weak gel and sol state for a 2% gellan solution in the presence of 10, 50, and 90 vol.% of oilfield water with salinity of 73 g∙L^−1^ [50].

The sol-gel and liquid–solid phase transitions of gellan in oilfield brine water of the Kumkol oil reservoir containing Na^+^, K^+^, Ca^2+^, and Mg^2+^ with a total salinity of 73 and 90 g∙L^−1^ was evaluated in dependence of the polymer concentration and temperature [46,47]. For 0.5 wt.% aqueous gellan solution, the addition of 10–40 vol.% oilfield saline water with a salinity of 73 g∙L^−1^ causes gel formation, while starting from 50 vol.% of oilfield saline water, a liquid and dense solid phase are formed (Figure 4a) [46]. Also, the addition of oilfield water with a salinity of 73 g∙L^−1^ to 2 wt.% gellan solution at first leads to the formation of bulk gel, which gradually transforms into the “weak gel”, and then into the sol state (Figure 4b) [50,51].

**Figure 5:**
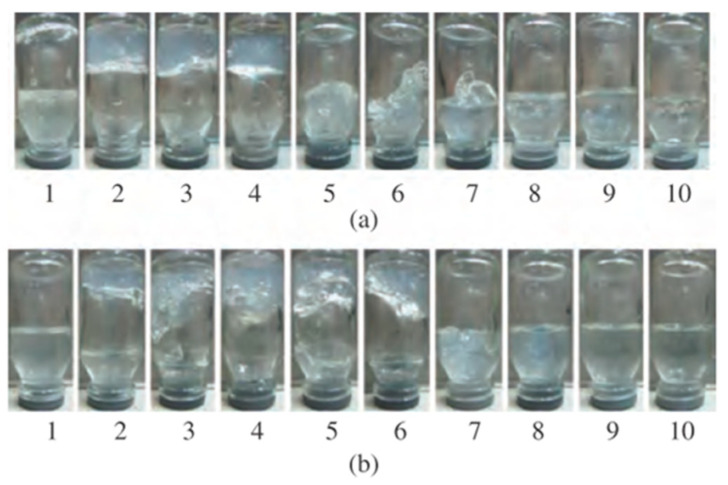
1.5 wt.% gellan solution gelation in oilfield water with salinity of 90 g∙L^−1^ at 30 °C (**a**) and 60 °C (**b**). The oilfield water content as follows: 0 (1), 10 (2), 20 (3), 30 (4), 40 (5), 50 (6), 60 (7), 70 (8), 80 (9), and 90 vol.% (10) [47].

For 1.5 wt.% aqueous solution of gellan, bulk gelation is observed upon the addition of 10–60 vol.% oilfield water with a salinity of 90 g∙L^−1^ irrespective of temperatures of 30 and 60 °C (Figure 5) [47]. While in the presence of >60 vol.% oilfield saline water, liquefaction of the system takes place.

The development of a frail gel and its subsequent transformation into a liquid state in high-saline water can be explained by the synergistic “salting out” effect of mono- and divalent cations. Whereas the initial formation of gel is explained by the acceleration of the charge-shielding effect by monovalent and the “bridging” effect by divalent ions. When combined, these factors contribute to the establishment of a double helix structure, further reinforced by hydrogen bonds [51]. It can be assumed that under very high salinity conditions, the “salting out” effect as well as the aggregation of the double stranded structure of gellan will result in its liquefication. However, as mentioned in the review [33], when divalent and monovalent cations are present together, as occurs in the case of oilfield water, the gelation mechanism of gellan, and the properties of the gels, can be complex and difficult to interpret. Nevertheless, the evaluation of the behavior of gellan in various oilfield water containing the mixture of mono- and divalent cations may be useful in selecting the required concentration of gellan for oil reservoirs with different salinities and temperatures.

**Figure 6:**
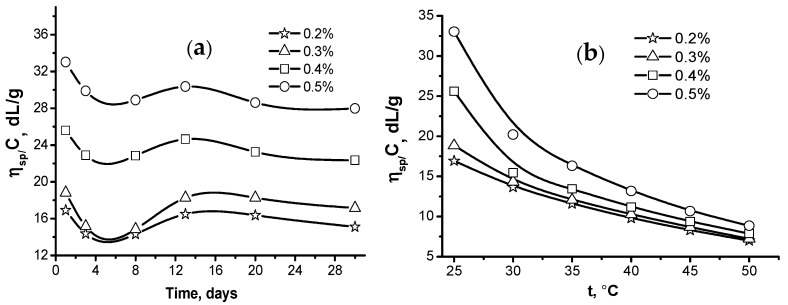
Storage time- (**a**) and temperature- (**b**) dependent reduced viscosity of gellan solutions [39,46].

The stability of the gellan solution with respect to the storage time and temperature was also studied [46]. The viscosity–time curves for 0.2–0.5% gellan solutions exhibit a similar trend (Figure 6a). The initial drop in viscosity is succeeded by a slight rise, which is then followed by subtle fluctuations. The lowest viscosity is observed after five days of storage. This temporal viscosity variation is likely associated with the formation and subsequent breakdown of associates held together by hydrogen bonds.

The viscosity of gellan solutions drops as the temperature rises (Figure 6b). The initial viscosities differ notably at 25 °C but converge at 50 °C. This is likely due to the gradual disintegration of macromolecular associations caused by the increasing temperature-induced destruction of hydrogen bonds.

**Figure 7:**
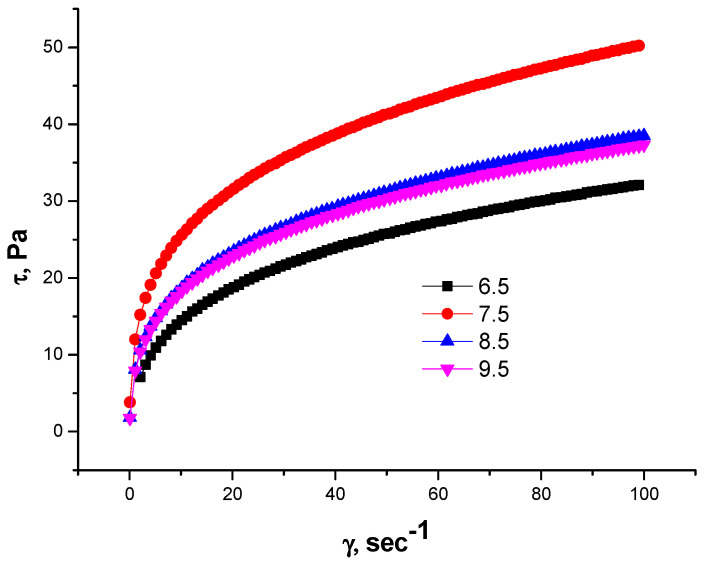
Dependences of shear stress (τ) on shear rate (γ) for 1.0 wt.% gellan solution at different pHs and T = 298 K [46].

The research endeavored to elucidate the impact of pH variations on the rheological characteristics exhibited by gellan solutions at concentrations of 0.5% and 1% [46]. At pH = 7.5, the 1.0 wt.% gellan solutions behave as shear-thinning fluids due to the ionization of glucuronic acid residues. At pHs of 8.5 and 9.5, the excess NaOH shields the glucuronate ions, plausibly attenuating the polyelectrolyte effect, and reduces the shear stress by shielding the macroions (Figure 7).

The strain–stress data of gellan gels induced by the addition of various salts including oilfield saline water as well as the Young’s modulus and breaking stress are shown in Table 5 [48].

**Figure 8:**
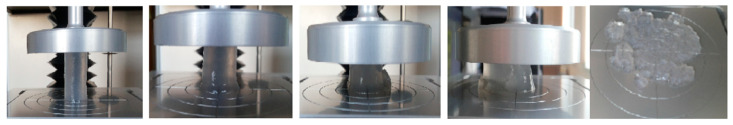
Compressing of gellan gel formed in 73 g∙L^−1^ oilfield brine [48].

Increasing the mechanical properties of gellan gel in the order: oilfield water > CaCl_2_ > MgCl_2_ > KCl > NaCl is in good agreement with rheological behavior. The enhanced strength of gellan gel in oilfield water, possibly due to the combined effect of monovalent and divalent cations in oilfield brine, is effective for plugging high-permeability channels in the oil reservoir (Figure 8).

## 3. Plugging Behavior of Gellan Gum in Laboratory Experiments

Our research team has conducted many laboratory tests on the plugging behavior of gellan gum [52,53,54]. Those tests include sand pack and core flooding, as well as fractured core flooding experiments. Aqueous solutions of gellan gum are able to reduce permeability by two mechanisms:

1- Gradual plugging due to the entrapment of particulate gel particles found in the gellan gum solution [55].
2- Instant gel formation as a result of gellan gum solution in contact with cations found in the brine saturating the porous rock or injected as a post-flush following the gellan gum slug [56].

**Figure 9:**
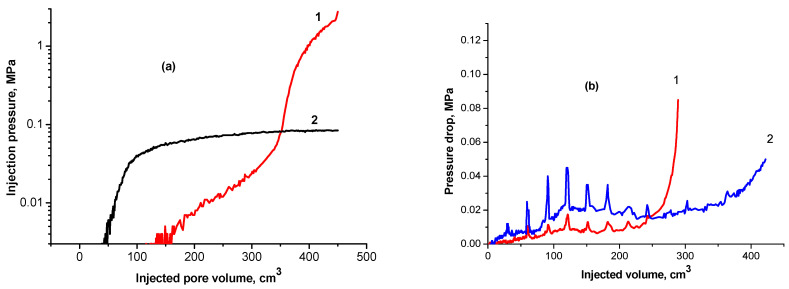
(**a**) Behaviors of 0.05 wt.% gellan gum (1) and polyacrylamide (2) solutions in porous media [45]; (**b**) pressure oscillating behavior of 0.1 wt.% gellan gum solutions in 3.80 Darcy (1) and 3.2 Darcy (2) at room temperature and fixed flux [52].

Figure 9a demonstrates the difference between gellan gum and polyacrylamide solutions’ behavior in porous media [53]. The polyacrylamide injection pressure grows up to a certain value, which depends on the rheology of the polymer solution, rock permeability, and the flux, and stabilizes. The pressure stabilization indicates no plugging inside of the porous media. At the same time, the 0.05 wt.% gellan gum solution demonstrates a gradual linear pressure increase followed by exponential growth, which indicates plugging. Figure 9b shows the remarkable pressure oscillating behavior of 0.1 wt.% gellan gum solution in 3.18 and 3.80 Darcy sand packs saturated with brine. Based on the experimental results obtained in [52,57], authors [53] developed the analytical model of oil recovery displacement via gellan penetration. This model takes into account the dependence of the viscosity of the polymer solution on the salinity, as well as the effect of polymer adsorption on the permeability, utilizing a computational algorithm in cylindrical coordinates. The proposed model was verified by comparing the numerical results obtained in the hydrodynamic simulator Eclipse 100 with experimental results.

The instant plugging of porous media by gellan gum injection was demonstrated with a core flooding experiment in which Kumkol oilfield (Kazakhstan) oil (well 2514, formation U-3) and brine (well 3064) were used [55,58,59]. The oil and brine viscosities at 55 °C (reservoir temperature) were equal to 1.91 and 0.80 cp, respectively. The total salinity of the brine was 71 g∙L^−1^. Two core samples from the same oilfield (well no. 2400, depth 1280–1289 m) were used, and their properties are listed in Table 6. The cores were connected together in the Hassler core holder sleeve to make a 10 cm-length compound sample. The core flooding was performed at 55 °C.

The core connate water and initial oil saturation were equal to 35.55% and 64.45%, respectively.

Oil displacement by water was conduction under a constant flow rate of 0.2 cm^3^/min. At the end of the water flooding, the water and irreducible oil saturation were equal to 50.95% and 49.05%, respectively. The oil recovery was equal to 23.89%.

**Figure 10:**
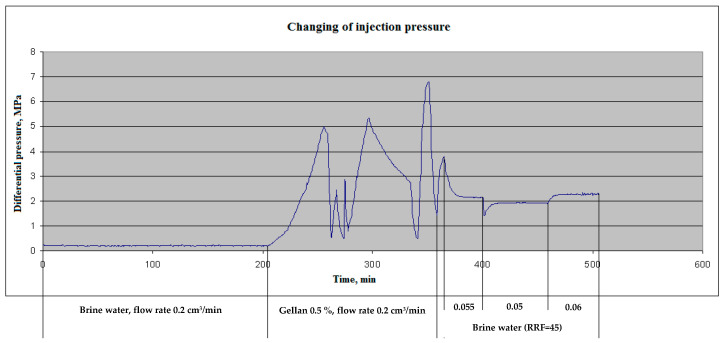
Injection pressure versus time. Compound 10 cm-length sandstone sample. Ambient temperature 55 °C.

After the water injection, 0.5 wt.% gellan gum solution was injected into the core at 0.2 cm^3^/min. Immediately after polymer injection, a dramatic increase in injection pressure was observed, and during the next 60–65 min, the pressure reached 5 MPa, and the probability of a Hassler core holder sleeve breakthrough appeared. The polymer injection was stopped to decrease the pressure down to an acceptable level and then was initiated again at the same flow rate. In this sequence, three cycles of polymer injection were performed. One pore volume of polymer was injected into the core samples. In Figure 10, three cycles of polymer injection are characterized with maximal injection pressure rises.

After 0.5 wt.% gellan injection, the reservoir brine post-flush was initiated at 0.2 cm^3^/min, but because of the rapid pressure increase, the flow rate was decreased to 0.055 cm^3^/min. After stabilization of the injection pressure (at 2.15 MPa) at 0.055 cm^3^·min^−1^, the flow rate was changed to 0.05 and then to 0.06 cm^3^·min^−1^. By making these alterations, the permeability of the plugged core sample was determined. The result showed that the initial permeability has been decreased by a factor of 45; i.e., the residual resistance factor (RRF) equals 45. (The RRF is defined as the brine differential pressure after the rock exposed to gellan divided by the brine differential pressure before exposure to the gellan [60,61]).

The final oil displacement after gellan injection was equal to 28.45%, which is 4.56% higher than water flooding.

In fact, both plugging mechanisms can be applied to reduce the permeability in the vicinity of a well; however, instant gelation of gellan gum solution upon its contact with the brine makes it a promising candidate for permeability reduction in fractures. If a polymer solution gels instantly once it enters the fracture, two problems will be solved:

1- Polymer extrusion occurring from the fracture interfaces into the matrix in the process of injection;
2- Slumping of the low viscosity (compared to gel) polymer solution due to the gravity of the lower part of the fracture.

Experiments on fracture models were used to demonstrate the dynamic behavior of gellan gum solution when it enters a fracture saturated with brine.

**Figure 11:**
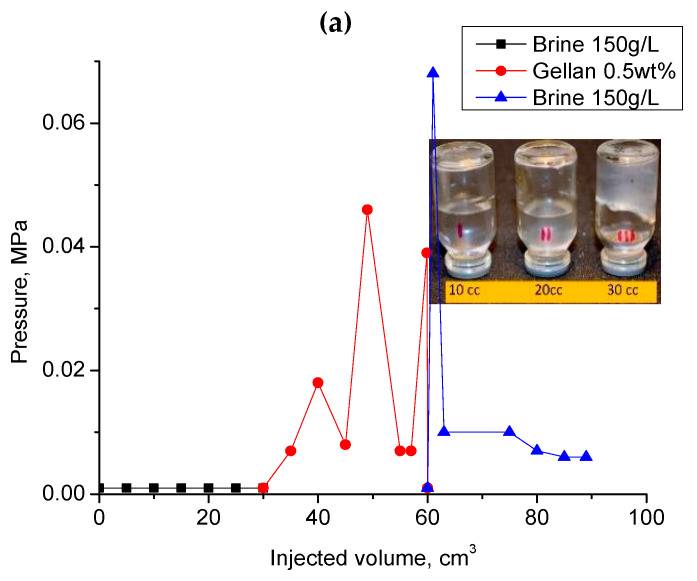

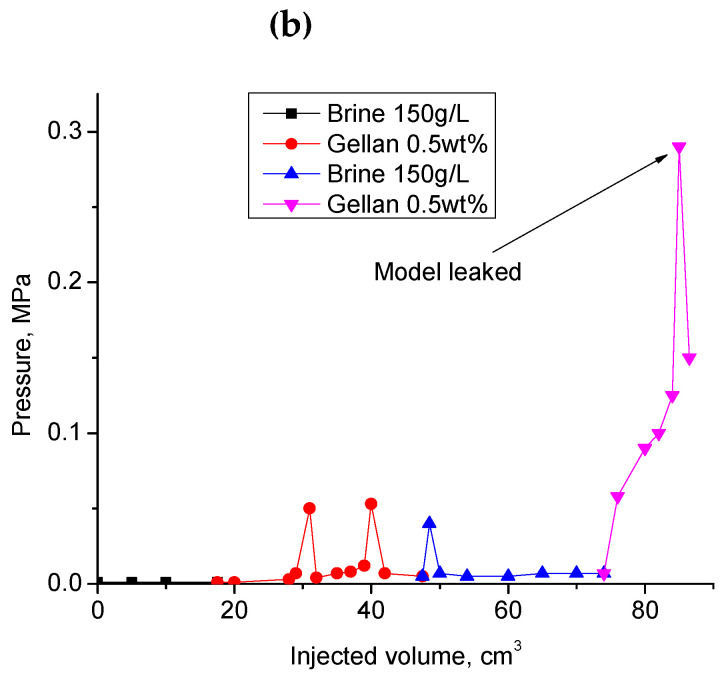
(**a**) Injection of 0.5 wt.% gellan gum solution into the 1 mm-height, 7 cm-width and 20 cm-length fracture model at 0.5 cm^3^·min^−1^ and 30 °C; (**b**) alternated injection of 150 g·L^−1^ brine and 0.5 wt.% gellan gum solution into the 1 mm-height, 7 cm-width and 0.2 m-length fracture-like feature at 0.5 cm^3^·min^−1^ and 30 °C. (**b**) demonstrates the 2nd cycle of alternated 0.5 wt.% gellan gum solution and 150 g/L brine injection into 1 mm-height fracture equipped with a sensor measuring pressure across the fracture right at its center.

Figure 11a demonstrates a significant injection pressure increase registered during the injection of 0.5 wt.% gellan gum solution into a 1 mm-height, 7 cm-width, and 20 cm-length fracture model. As can be seen, the continuous injection of gellan gum solution resulted in the displacement of gel from the fracture, which was accompanied by a pressure decrease. The following brine post-flush caused a sudden pressure increase. This may indicate a gellan gum rear front gelation initiated by the introduction of brine into the fracture followed by brine breakthrough indicated by a sharp pressure decline. This result implies that alternated injection of gellan gum and brine will allow maximal gel concentration in the porous media or fracture. For example, Figure 11b demonstrates that during the second gellan gum injection step, which was initiated after brine post-flush, the inlet pressure grows up to 0.275 MPa before the model leaks. This gives a pressure gradient of 1.375 MPa/m (0.275 MPa/0.2 m) or 60 psi/ft, which is a relatively high value, since 0.5 wt.% mature hydrolyzed polyacrylamide gels require around 0.45 MPa/m (20 psi/ft) to be extruded through 1 mm-width fractures [62].

During the second gellan gum slug injection, the inlet and center pressure values became equal. This indicates that the total volume of the fracture was filled with gellan gel. The following post-flush with 150 g∙L^−1^ brine was also characterized by almost equal inlet and center pressure values. The increase in the flow rate by 20 times resulted in the model leak at 0.2 MPa.

**Figure 12:**
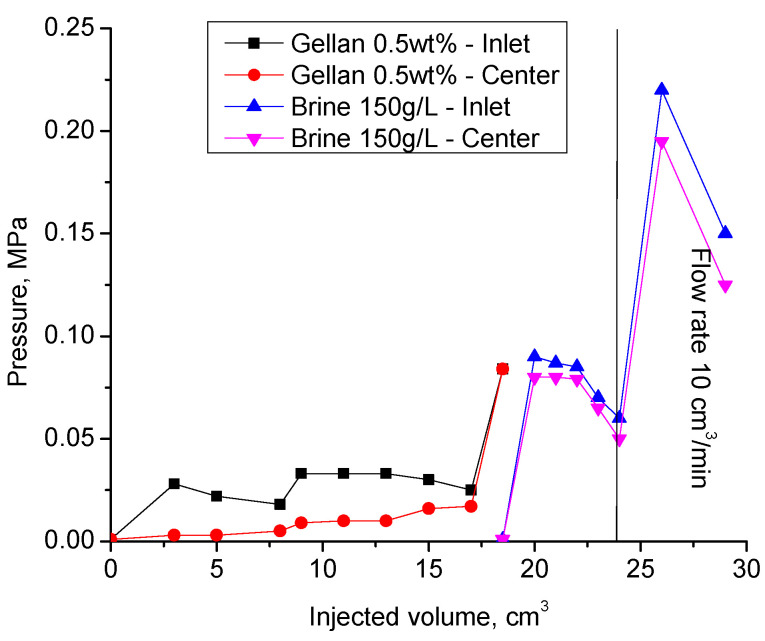
Results of the second cycle of alternated gellan gum solution and 150 g·L^−1^ brine injection into the 1 mm-height, 7 cm-width, and 20 cm-length fracture model at 30 °C. Previously, the fracture was flooded with the same volume of gellan and brine (1st cycle).

Of importance is that the experiments shown in Figure 11 and Figure 12 were conducted by using fractures with an impermeable matrix, which is not relevant to conventional oil reservoirs. More work needs to be completed in order to understand the plugging behavior of gellan gum solutions in fractured cores. Anyway, the experimental results presented above prove that gellan gum provides sufficiently high-pressure gradients (around 50 psi/ft = 1 MPa/m = 0.2 MPa/0.2 m) in 1 mm-width fractures during the post-flush with high salinity brine (150 g/L) (Figure 12). This is a remarkable result because post-flush brine salinity greatly influences the stability of HPAM gels in fractures [63]. For example, the authors of [64] found out that HPAM + Cr^+3^ gel failed at 0.45 MPa/m (20 psi/ft) during the post-flush with 8% salinity brine; as a consequence, there was no redirection of fluids accomplished. Moreover, the literature analysis of other authors’ laboratory studies [62,65,66,67,68] has shown that the resistance of HPAM gels to wash out with brine reduces with an increase in the fracture width according to the following exponential law:

$$\frac{dp}{dl}=75.46e^{-2.128x}$$

where *dp*/*dl*—pressure gradient required for the brine to break through the gel plug in the fracture, psi/ft; *x*—fracture width, mm.

**Figure 13:**
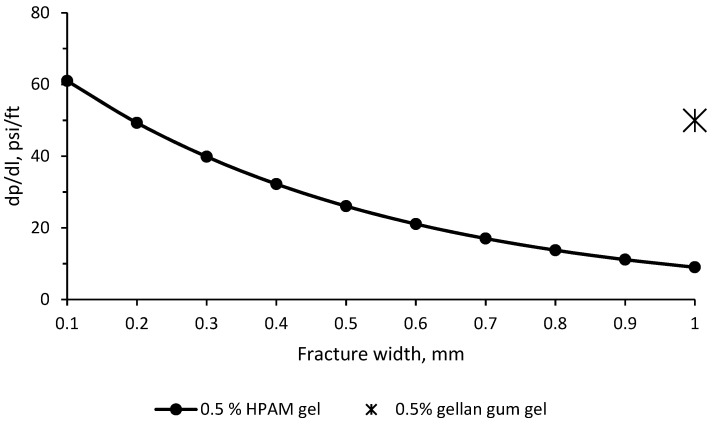
Pressure gradients provided by HPAM (∙) or gellan gum (∗) gels inside of a fracture during the post-flush.

The equation presented above was used to compare the plugging efficiency of HPAM gel with that of gellan gum gel. Figure 13 below shows that in a 1 mm-width fracture, HPAM gel is not expected to provide higher than 0.45 MPa/m (20 psi/ft) during the post-flush, whereas gellan gum gel provides 1.13 MPa/m (50 psi/ft). Thus, the pressure gradient observed during the water post-flush after gellan gum injection is 2.5 times greater than that observed with HPAM gel, signifying superior plugging efficiency. This substantial contrast can be attributed to the enhanced gel strength and viscosity associated with the unique gel structure discussed in Section 2 earlier. In fact, the results presented in [55] show that the bulk gel strength of gellan gum was in the range of 0.1–0.22 MPa, whereas HPAM gels demonstrated 0.0017–0.0225 MPa under similar conditions.

**Figure 14:**
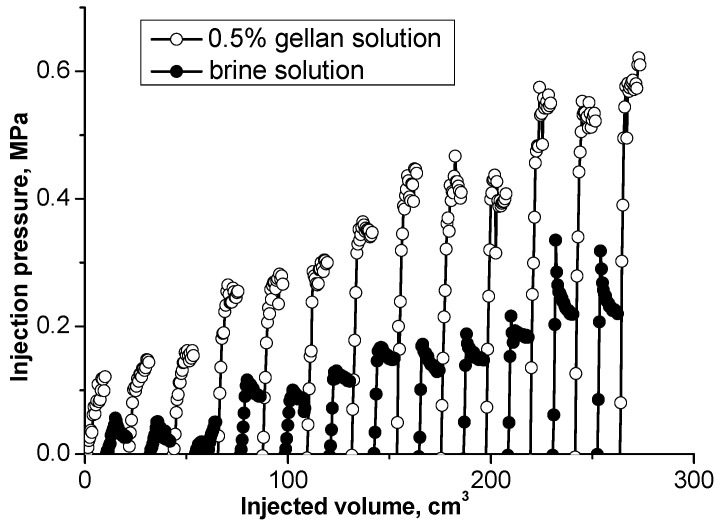
Injecting 0.5 wt.% gellan and brine solutions slugs into a high permeability sand pack [54].

The results presented in [54] demonstrate that alternated injection of gellan gum solution and brine into a sand pack model leads to a gradual inlet pressure increase (Figure 14). This approach may be used to plug high-permeability channels in unconsolidated sand reservoirs.

Alternated injection of gellan gum with brine into a 1 mm-fracture model is described in [55]. The results show that three cycles of gellan–brine injection were required to achieve a maximal brine injection pressure equal to 0.5 MPa at the fracture inlet. Whereas the maximal brine post-flush pressure registered at the fracture inlet after HPAM mature gel injection was equal to only 0.083 MPa. These results support the application of gellan gum for permeability reduction in fractures.

**Figure 15:**
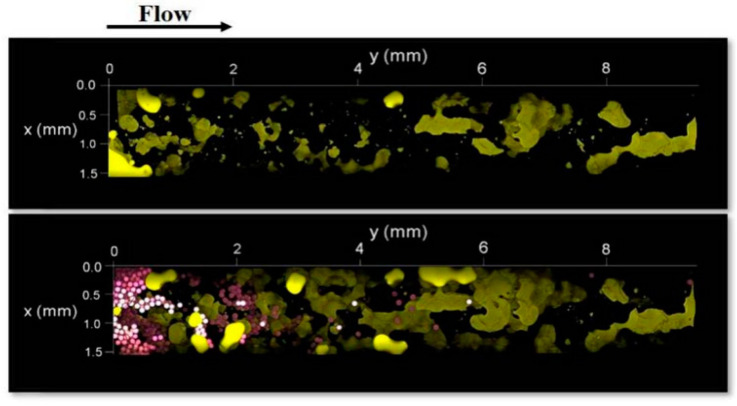
Bottom views of the heterogeneous porous medium before (**upper**) and after (**lower**) the injection of aqueous phase with microcapsules. Yellow—oil; purple—microcapsules [69]. Published with permission from Society of Petroleum Engineers. License ID—1338897-1.

Almost in all tests, the gellan gum was injected in the form of a solution; however, in [69], the gellan gum was used to prepare 104 μm-diameter microcapsules with a soft 5 μm-thickness shell, which presumably allowed for better penetration into 120–200 μm-size pores [69]. The injection of the microcapsules into 38%-porosity and 232D-permeability homogeneous porous media made of glass spheres allowed for the mobilization of residual oil saturation (immobile oil), which was visually proven by using confocal microscopy (Figure 15). In fact, it was shown that within an 11 mm-length porous model, the injection of microcapsules allowed for a reduction in the residual oil saturation from 32.9 to 25.7% [69]. Unfortunately, the images presented in the paper demonstrate that even in high-permeability porous channels, the deep penetration of microcapsules is problematic. Moreover, no pressure gradient data were presented in this paper; however, from the figure above, it is evident that the gel microcapsules plug the porous media.

## 4. Treatment of Injection and Production Wells by Gellan Gum

### 4.1. Injection Well Treatment

What can be expected from gellan gum solution if it is injected into an injection well to improve conformance control?

The papers [55,58,59,70] present the results of 160 and 234 m^3^ of gellan gum injection into two injection wells at Kumkol oilfield (Kazakhstan). As a result, 3790 tons of oil were produced incrementally in 6 months. Further monitoring showed that 5890 tons (43,940 bbls) of oil were produced incrementally in 11 months. The calculated IOR equals 3.52 m^3^ (22 bbls) of oil per 1 kg of dry polymer; in total, 1 ton of dry polymer powder was injected in each well. The IOR is the improved oil recovery or additional oil recovery due to the influence of gellan injection in our case.

**Figure 16:**
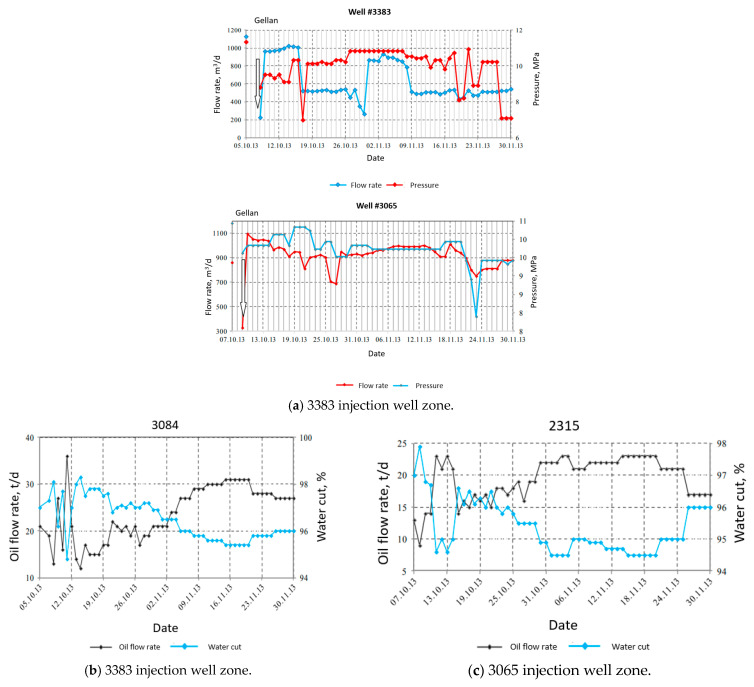
Water injection pressure and flow rate after the gellan injection into wells 3383 and 3065 on 6–8 October 2013 (**a**) [71]. Production wells response to gellan injection (**b**,**c**) [71].

Of importance is that the wellhead pressure increased from 60 to 90 atm during the injection of gellan gum, which indicates permeability reduction due to polymer gelation. Both plots in Figure 16 demonstrate that the initial water injection pressure increase, observed right after the gellan injection, was followed by its reduction. This is explained by the gellan slug rear front gelation in high permeability zones and the oil driven by the injected water from the lower permeability zones. The increase in the oil flow rate and the water cut reduction at some of the offset production wells proves that additional oil was mobilized after the treatment (Figure 16). The water cut is defined as the ratio of the water volume that is produced in a well compared to the volume of the total liquids produced.

Below, the results of the injection well treatment presented in different literature sources are provided for the purpose of comparison.

The authors of [72] analyzed the results of 114 injection well HPAM or xanthan gel treatment projects conducted in different reservoirs in a wide range of conditions. The results show that the projected IOR varied from 0 to 200 m^3^/kg (560 bbl/lb) of polymer with an average value of 0.85 m^3^/kg (2.4 bbl/lb) of polymer.

In one of the largest Latinoamerican polymer gel projects, 6857 m^3^ (43,400 bbl) of gelant (polymer and crosslinker solution) was injected into the injection well. In 4 years and 7 months, 54,400 m^3^ (340,000 bbls) of incremental oil recovery was achieved. Taking into account that the average polymer concentration was around 5000 ppm, approximately 34 tons of dry HPAM were injected into the well. This makes 1.6 m^3^/kg (10 bbls/kg) of polymer [73].

In the Balcon field, two injectors characterized by severe water channeling, high injectivity, and low secondary oil recovery were treated in 2010 by injecting 4774 m^3^ (29,840 bbls) and 1323 m^3^ (8269 bbls) of gelant. In total, around 16,680 kg of dry HPAM polymer were injected. A total of 33,920 m^3^ (212,000 bbls) of oil was recovered at an increment of 10 months. Thus, 2 m^3^/kg (12.7 bbls/kg) of polymer was produced incrementally in 10 months [74].

The authors of [75] analyzed the results of 61 gel treatment field projects and found out that typical incremental oil production equals 3.5 m^3^/kg of polymer (10 bbl/lb), which is exactly the number achieved in the Kumkol gellan injection project.

One of the most successful fractured injection well gel treatments was performed in the Big Horn Basin. The first 17 well treatments allowed for the recovery of 584,000 m^3^ (3650,000 bbls) of incremental oil. On average, this makes 34,400 m^3^ (215,000 bbls) of incremental oil per gel treatment. Stated in another way, the IOR equaled to 4.8 m^3^/kg of polymer (13.6 bbl/lb of polymer) [76]. Table 7 summarizes the above-mentioned projects.

As seen from Table 7, among the six reviewed cases, the gellan gum in the Kumkol project occupies the second place in terms of incremental oil recovery per 1 kg of dry polymer injected.

### 4.2. Production Well Treatment

**Figure 17:**
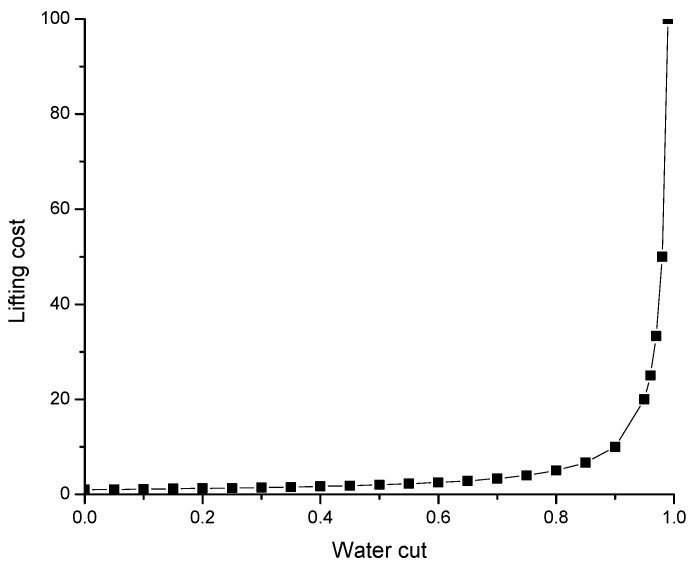
Oil lifting cost versus water cut [60].

The value of polymer/gel injections into production wells is not only in the possibility of an oil production increase. The reduction in costly and environmentally unfriendly water production is also important. For example, 300 gel treatments conducted in the Arbuckle Field allowed for shut off of 17.6 million m^3^ (110 million bbls) of water production [76]. Of importance is that the lifting cost of oil increases exponentially with the increase in water cut beyond 90% (Figure 17) [60]. That is why a decrease in water cut by a few percent within the range between 100 and 90% can substantially reduce the oil lifting cost.

The injection of polymer and gels into the production wells can significantly reduce the water/oil ratio and in some cases increase the oil flow rate unless the oil-producing zones are damaged by the polymer/gel.

**Figure 18:**
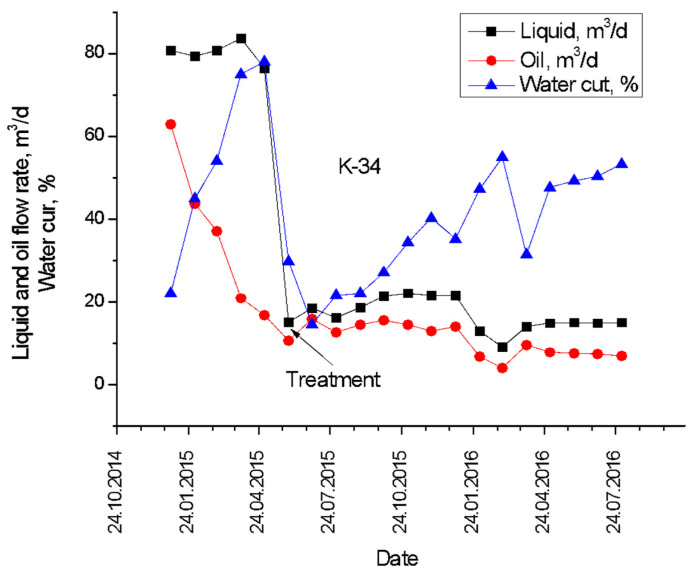
Pre- and post-treatment production history of Karabulak producing well K-34 [59].

In June 2015, producing well K-34 at Karabulak oilfield (Kazakhstan) was treated with 120 m^3^ of gellan gum solution with an average concentration of 1 wt.% in order to reduce the water cut, which by that time increased up to 77–80% (Figure 18) [59]. Immediately after the treatment, the water cut reduced to 20%, whereas over the next year, the water cut increased up to 60%. At the same time, it is remarkable that the oil production rate was not very much affected by the gel injection.

The comparison of the Karabulak well treatment results (Figure 18) with others published in the literature (Table 8) proves the effectiveness of using gellan gum for the treatment of production wells.

## 5. Challenges and Opportunities of Gellan Gum Application in Field Conditions

This section summarizes the primary advantages and disadvantages of utilizing gellan gum in comparison to HPAM, which is currently the most common EOR polymer.

Primary advantages of gellan gum:

1- The crosslinking chemicals are not needed;
2- High gel strength in high brine salinity conditions;
3- A relatively low dissolution time (30 min) is required.

Primary disadvantages of gellan gum:

1- The reagent should be dissolved in fresh water (no more than 1 g/L);
2- High price (8–20 USD per 1 kg);
3- Cannot be used for polymer flooding due to the presence of microgel particles in the solution.

Primary advantages of HPAM:

1- Low price (2.5–4 USD per 1 kg);
2- The high elasticity of gels allows for injection of large volumes at an acceptable pressure;
3- Can be used for polymer flooding.

Primary disadvantages of HPAM:

1- The crosslinking chemicals are needed;
2- The gel is not stable upon contact with high-salinity water;
3- High polymer adsorption in carbonate rocks;
4- Relatively high dissolution time is required (2–3 h).

Based on the information presented above, when there is an ample supply of fresh water and the budget is not restricted to 4 USD/kg of dry powder, gellan gum may prove to be a preferable alternative to HPAM. Furthermore, the samples of gellan gum gels obtained during the Kumkol pilot test (2013) were aged for 5 years without any evidence of gel degradation [63]. In reservoir conditions, microbial degradation of biopolymers may also be prevented through the appropriate use of biocides [80].

The rapid gelling ability of gellan gum upon contact with reservoir brine presents several opportunities for its application either as a standalone component or in combination with HPAM. Prior studies have demonstrated that HPAM gel placed in a fracture fails to divert 80,000 ppm brine into the matrix at a pressure gradient of 0.45 MPa/m (20 psi/ft). In contrast, when low-salinity water is injected, a 100% diversion is observed at 2.26 MPa/m (100 psi/ft) [64]. To enhance the performance of HPAM gels under high-salinity conditions, it is recommended to inject a gellan gum slug during the final stage of treatment. This will create a barrier safeguarding the rear edge of the HPAM gel from direct contact with the high brine salinity near the well where pressure gradients are high.

Another opportunity to reduce the quantity of expensive gellan gum, potentially by half, is to inject it alternately with high-salinity brine in a 1:1 ratio [54].

Furthermore, there is potential for partial substitution of gellan gum with cost-effective filling materials, such as fibers, wood dust, and particulates. This approach can enhance gel strength and decrease the overall treatment cost.

## 6. Economical Feasibility of Gellan Gum in EOR over Other Technologies

Based on the available data [81], 1218 tons of HPAM were utilized, resulting in a notable 76.6-ton increase in oil production for every ton of polymer employed. This led to an almost doubling of oil production, coupled with an approximately 10% reduction in water cuts as observed [82]. The efficiencies of different technologies applied for EOR, including gellan, are compared in Table 9. Even though all these pilot tests were conducted under varying reservoir conditions, it is important to note that the mass of gellan gum used ranged from 2 to 5 tons, resulting in a technological efficiency range of 1160 to 2945. In contrast, the mass of HPAM used ranged from 16 to 42 tons, leading to a technological efficiency range of only 380 to 1703.

In the world market, the price of commercial HPAM is varied in the range of 3–5 USD per 1 kg, while the price of commercial food gellan in the world market is 15–20 USD per 1 kg depending on the purity and quality. Taking into account that in Kazakhstan, the average additional oil recovery is 76.6 tons of oil per 1 ton of HPAM [81], while, according to data in Table 9, 1 ton of gellan is able to produce from 1000 to 3000 tons of additional oil during 6–11 months, one can evaluate the efficiency of two reagents. Simple calculations show that the polymer utilization factor of gellan is roughly 10–40 times higher than that of HPAM, while the price of 1 kg of gellan is only 3–6 times higher than HPAM. These figures clearly indicate that the use of gellan for oil recovery is much more effective than HPAM.

## 7. The Potential for Gellan Production in Kazakhstan

**Figure 19:**
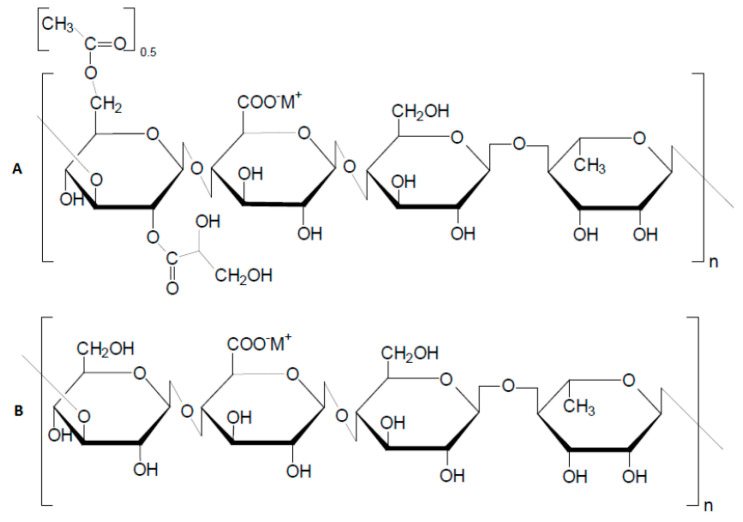
Structure of repeating monomeric units of native high-acyl gellan gum (**A**) and deacylated or low-acyl gellan gum (**B**) [82,83].

The Institute of Polymer Materials and Technology (www.ipmt.kz, accessed on 1 January 2023) research team, in partnership with the biotechnology lab at Kazakh National University, has recently succeeded in gellan production technology by using glucose–fructose syrup sourced from Zharkent and Burunday corn starch plants (krahmalopatoka.kz) [88]. The fermentation of these substances by Sphingomonas paucimobilis ATCC^®^ 31461 results in the production of both high- and low-acyl gellan (HAG and LAG), as detailed in references [89,90] (Figure 19).

**Figure 20:**
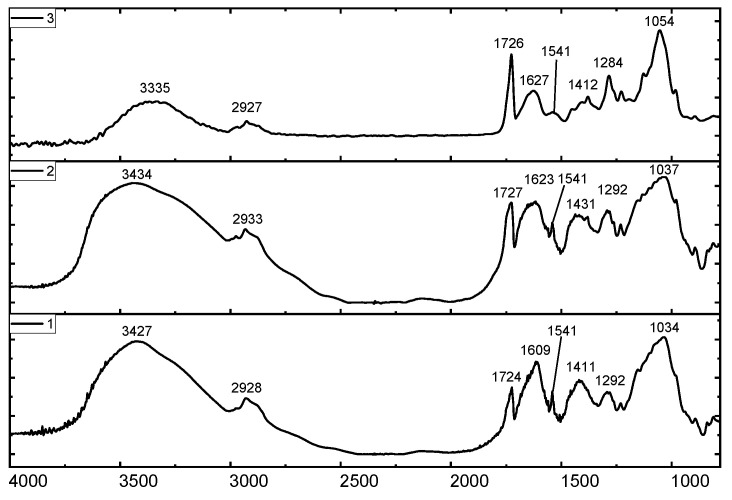
FTIR Spectra: self-purified (1) and technical (2) HAG manufactured by Xinjiang Fufeng Biotechnologies Co., Ltd., contrasted with fermentation-derived Kazakhstan HAG (3).

The primary distinction between the high- and low-acyl gellans is that the former is presented with the acyl group at O (2) and the glyceride at O (6). These groups are indicated by the intensive absorption band at 1726 cm^−1^. The FTIR spectra of commercial HAG manufactured by “Xinjiang Fufeng Biotechnologies Co., Ltd.” (Xinjiang Wurumqi City, China) and Kazakhstan HAG derived from the fermentation broth are shown in Figure 20. In all cases, the appearance of intensive bands in the range of 1724–1727 cm^−1^ is specific for acyl groups of HAG.

In deacylated gellan gum, or LAG, the prominent peaks in the range of 1724–1727 cm^−1^ vanish, confirming the removal of the acyl groups from HAG. Both the commercial and Kazakhstan LAG exhibit characteristic bands at 3340, 1406, 2921, 1622, and 1037 cm^−1^, corresponding to OH stretching and bending, CH stretching, C=O, and C-O-C bonds, respectively.

**Figure 21:**
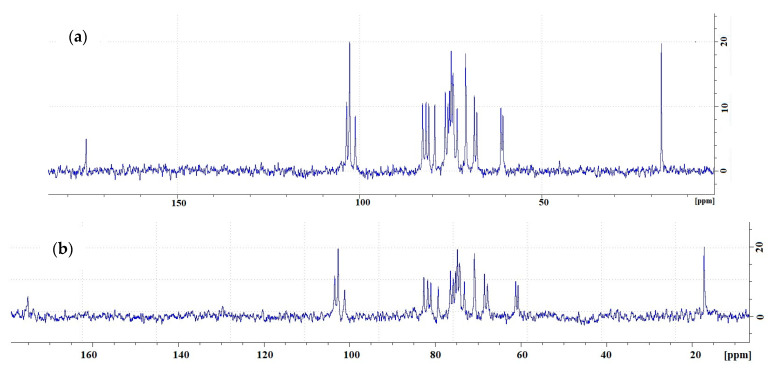
Carbon-13 nuclear magnetic resonance spectra of commercially produced (**a**) and Kazakhstan (**b**) low-acyl gellan at 60 °C.

The ^1^H NMR spectra of LAG are in good agreement with the previous results [46] and display the specific peaks of CH of rhamnose (5.27 ppm), CH of glucuronic acid (5.09 ppm), and CH_3_ of rhamnose (1.86 ppm). As Figure 21 shows, the ^13^C NMR spectra of commercial LAG and Kazakhstan LAG are in good agreement.

The thermogravimetric curves (TG) for both the commercial gellan and the HAG produced in our research exhibit close correspondence, with both samples undergoing decomposition around 250 °C (TG curves omitted).

Table 10 presents the weight–average molecular weight (Mw), number–average molecular weight (Mn), and polydispersity index (PDI) of HAG, as determined by GPC.

The dynamic viscosities of the biomass from various raw materials follow this order of increase: Burunday glucose–fructose syrup > Zharkent glucose–fructose syrup > glucose. This indicates that the biomass derived from Burunday glucose–fructose syrup is better suited for both HAG production and oil recovery (see Table 11).

**Figure 22:**
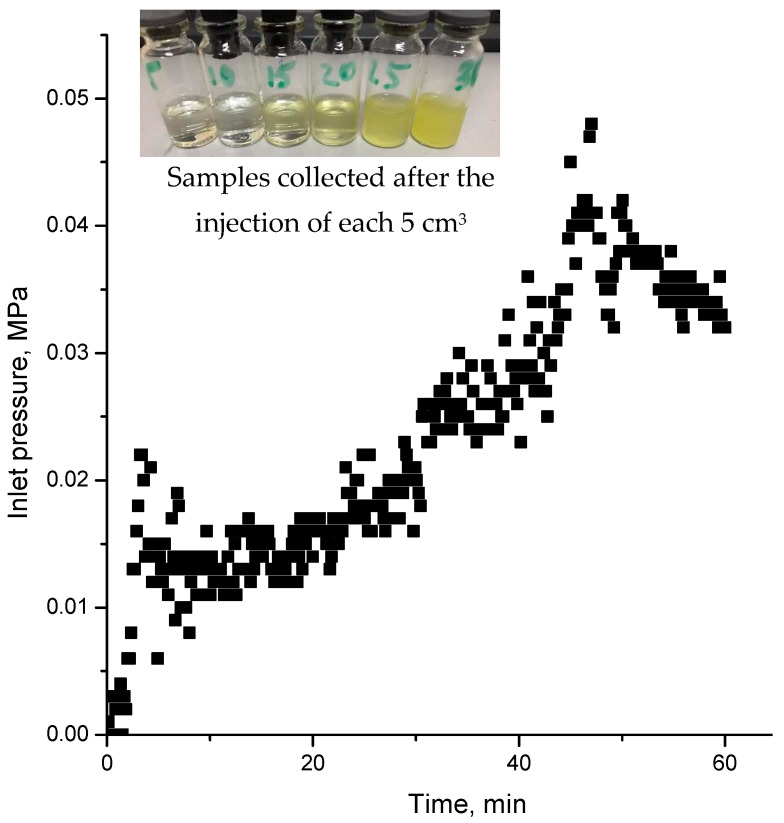
Injection pressure versus time registered during the injection of biomass into sand pack model. Insert is effluent samples collected after injection of 30 cm^3^ of biomass.

Aqueous solutions of 0.25% and 0.5% Kazakhstan LAG demonstrated impressive gelation properties with the introduction of 0.01–1.0 M NaCl and/or CaCl_2_. Ref. [22] previously illustrated the potential application of HAG in EOR. In our approach, we directly injected the fermented biomass acquired from Burunday glucose–fructose syrup, which exhibited a dynamic viscosity of 4420 mPa∙s, into the sand pack model without the need for further purification from a nutrient medium and other ingredients (refer to Figure 22). It was expected that in case of positive results, the direct injection of the biomass into the reservoir would make it possible to organize the technology for the production of gellan directly in the fields.

The steady rise in pressure is likely attributed to the gradual plugging of pores by fine gel particles, which are screened out at the inlet. The effluent samples contain fine gel particles originating from the biomass.

**Figure 23:**
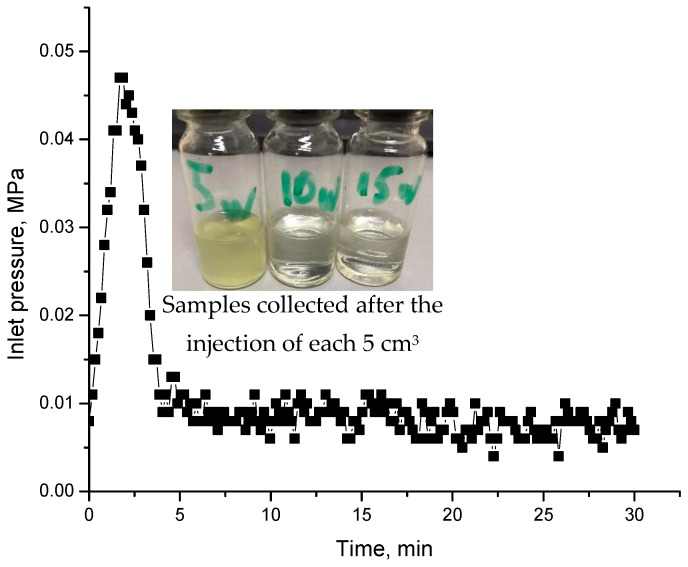
Injection pressure versus time registered during the injection of post-flush. Inserts are effluent samples collected after injection of 15 mL of water.

Post-flushing the model with water results in a sudden increase in pressure explained by the displacement of gel particles by water (Figure 23).

These experiments demonstrate that the fermented biomass was capable of moderately reducing the permeability of the sand pack model. However, further testing and optimization are required to make this product suitable for treating conformance control issues in oil reservoirs.

## 8. The Use of Gellan Gum in Food Industry, Biotechnology, and Medicine

Polysaccharides, such as gellan, hold significant potential in various fields, extending beyond enhanced oil recovery (EOR) applications [91,92,93]. In fact, gellan is a widely utilized polysaccharide in the food industry [94], biotechnology [95], medicine [96], pharmacy [97], and tissue engineering [98]. Its specific gelling properties in different mediums have led to the development of controlled release forms, including oral, ophthalmic, nasal, and others [99]. Gellan gum-based hydrogels demonstrate excellent in vivo and in vitro biocompatibility [100], with adjustable physical and mechanical properties suitable for applications in cartilage regeneration [101,102], cell encapsulation [103], nucleus pulposus regeneration [104], and more. Recent advancements in the application of gellan in biotechnology, medicine, and pharmacy are thoroughly discussed and summarized in review [90].

## 9. Conclusions

In this article, the applicability of gellan gum for addressing conformance control issues is reviewed by drawing upon the available literature data. The following conclusions can be made:

The sequence for the efficacy of salts in augmenting gellan gelation is as follows: BaCl_2_ > CaCl_2_ ≈ MgCl_2_ > KCl > NaCl.

The specific heat capacity (∆C) of 0.5 wt.% gellan solution at salt concentrations of 0.005 and 0.01 mol∙L^−1^ changes in the following sequence: CaCl_2_ > MgCl_2_ > KCl > NaCl and coincides well with the viscometric results. However, this sequence was not performed at salt concentrations 0.05 and 0.1 mol∙L^−1^.

The ability of gellan solution to gel upon contact with brine with a total salinity of 73 and 90 g∙L^−1^ was evaluated for different temperatures and polymer concentrations. The obtained results may be useful in selecting the required concentration of gellan for a field trial.

The viscosity of 0.2–0.5 wt.% gellan solution was stable for 28 days. Monotonic decreasing of the reduced viscosities of diluted gellan solutions upon increasing the temperature may be explained by the gradual disaggregation of macromolecular associates due to the destruction of hydrogen bonds.

The rheological behavior of 0.5% and 1% gellan solutions indicates that maximal shear stress occurs at a pH of 7.5 when the gellan macromolecules expand due to glucuronic residue ionization.

In the core flooding tests, alternating injections of gellan gum and brine effectively filled a 1 mm-width fracture with gel. In fact, the gellan gum provided a ~6 times higher resistance to the brine flow in a 1 mm-width fracture compared to the HPAM gel. These results demonstrate the effectiveness of gellan gum in reducing permeability in fractures.

The results of the injection well tests at Kumkol oilfield in Kazakhstan show that 1 kg of dry gellan allows for the incremental production of 3.5 m^3^ (22 bbls) of oil. The treatment of the production wells with a 1 wt.% gellan solution resulted in a considerable decrease in water cut, from 80% to 10–20%, without affecting the oil flow rate.

High- and low-acyl gellan gums were derived through the fermentation of glucose–fructose syrup from Zharkent and Burunday corn starch plants using *Sphingomonas paucimobilis*. They were compared with a commercial gellan produced in China. In perspective, the developed technology of gellan production from the domestic raw materials of Kazakhstan may be scaled up and used in the food and oil industries. It is also expected that the direct injection of biomass into the reservoir would make it possible to organize the technology for the production of gellan directly in the fields.

Despite the aforementioned advantages, the higher price of gellan gum compared to HPAM limits its use in the oil industry. However, if attempts to reduce the manufacturing cost of polysaccharides are successful, they will become more competitive.

## Figures and Tables

**Table 1:** Thermal and duration stability of xanthan, scleroglucan, diutan, and HPAM in saline water [26].

Polymer	Salinity, ppm	T, °C	Time, Days	Refs.
Xanthan	170 000	80	300	[27]
Scleroglucan	30 000	100	720	[12]
	-	80	90	[28]
	-	90	-	[29,30]
Diutan	-	85	90	[1]
HPAM	30 000	85	100	[31]
HPAM	20 000	90	619	[32]

**Table 2:** Metal ion content of commercial sample of gellan gum.

Type of Metal Ions	K^+^	Na^+^	Ca^2+^	Mg^2+^	Cu^2+^	Zn^2+^	Pb^2+^
Content, meq∙g^−1^	0.671	0.41	0.32	0.21	0.016	0.008	<0.001

**Table 3:** The critical concentration of salts inducing sol-gel transition of 0.2 wt.% gellan solution.

Salt Type	BaCl_2_	CaCl_2_	MgCl_2_	KCl	NaCl
C_crit_ inducing the sol-gel transition of gellan derived from viscometric data illustrated in Figure 1b, mol∙L^−1^	0.0045	0.006	0.006	0.01	0.1

**Table 4:** The specific heat capacity (∆C) of 0.5 wt.% gellan solution with added salts [46].

Salt Type	∆C *, J∙g^−1^∙K^−1^			
	Concentration of Salt, mol∙L^−1^			
	0.005	0.01	0.05	0.1
NaCl	107.63	95.58	66.33	59.93
KCl	111.75	105.70	103.23	96.07
CaCl_2_	113.47	113.28	100.64	84.09
MgCl_2_	118.90	117.38	96.50	92.38

* The specific heat capacity (∆C) of 0.5 wt.% pristine gellan solution is equal to 129.71 J∙g^−1^∙K^−1^.

**Table 5:** Mechanical characteristics of gellan gel obtained by the addition of different salts and oilfield brine [48].

Type of Salts	Young’s Modulus, 10^−2^ N·m^−2^	Fracture Stress, %
NaCl	7.84	26.1
KCl	8.45	31.7
MgCl_2_	9.01	31.7
CaCl_2_	9.36	32.0
Oilfield saline water	9.54	33.1

**Table 6:** Main characteristics of rock samples.

Sample Number	Length, cm	Diameter, cm	Gas Permeability, mD	Porosity, %	Pore Volume, cm^3^
28	5.07	2.39	203.17	21.97	5.564
24	5.07	2.5	66.71	18.3	4.661

**Table 7:** Results of injection well gel treatments.

Project and Source of Data	m^3^ (bbls) of Incremental Oil Produced per 1 kg of Dry Polymer Injected, m^3^ (bbls)/kg	Refs
Average result of 114 injection well treatments with HPAM or xanthan gels *	0.84 (5.28)	[72]
Tello Field Pilot, 1 injector, HPAM gels	1.6 (10)	[73]
Balcon field 2 injectors, HPAM gels	2 (12.7)	[74]
61 gel treatment filed projects	3.52 (22)	[75]
Kumkol, 2 injectors, gellan gum	3.52 (22)	[52]
Big Horn Basin, 17 injectors, HPAM gels	4.78 (29.92)	[76]

* Most results are projections and were published near the start of a project.

**Table 8:** Results of production well treatment with polymer gels.

Treatment	Oil Production Increase	Water Cut Decrease	Literature Source
274 production well polymer and gel treatments	On average 3 times increase	On average from 98.7 to 87.5% (by 11%)	[72]
768 m^3^ of HPAM gel into the horizontal with water coning	From 0 to 16.3–27.4 t/d	Decrease by 6–9% on average within one year following the treatment	[77]
500 m^3^ of HPAM gel into the horizontal with water coning	From 9.2 to 20.0 m^3^/d	Reduction from 90 to 8%. Increased to 60–70% over the next 14 months.	[78]
Horizontal well, above an aquifer in a fractured carbonate reservoir. 90 m^3^ of microgel slug alternated with 50 m^3^ of polymer gelant	From 6.4 to 14.4 m^3^/day	Reduction by 11%	[79]

**Table 9:** Comparison of the efficiency of various EOR technologies.

Oilfield/Reagent, Year	Number of Injection Oil Reservoirs	Mass of Dry Reagent, Tons	Mass of Additional Produced Oil, Tons	Polymer Utilization Factor *, Tons/Tons	Duration, Months	Literature Source
Kumkol, Kazakhstan/Gellan gum, 2013	2	2	5890	2945	11	[58]
Kumkol, Kazakhstan/Gellan gum, 2014	3	3	8695	2898	8	[83]
Balcon, Colombia/HPAM, 2010	2	16.68	28,418	1703	10	[74]
Tello, Colombia/HPAM, 2009	1	34	45,576	1340	55	[73]
Kumkol, Kazakhstan/Gellan gum, 2017	5	5	5808	1160	6	[83]
Buzachi, Kazakhstan/Polyacrylamide,2011	1	42	16,000	380	12	[84]
Daqing, China/Gel–polymer system	4	134	15,000	113	10	[85]
Zhong-guang, China/Gel–polymer system	2	20.5	3239	158	3	[86]
Usinskoe, Russia/Thermotropic gel “Galka”	No data	117	10,316	88	6	[87]

* Polymer utilization factor is the ratio of the amount of additionally produced oil to the amount of the reagent used.

**Table 10:** Molecular weights and PDI of Kazakhstan HAG derived from glucose–fructose syrup of Zharkent corn starch plant.

HAG Produced from Glucose–Fructose Syrup of Zharkent Corn Starch Plant	Molecular Mass, Dalton			PDI
	M_w_	M_n_	M_z_	M_w_/M_n_
	343,500	333,000	360,000	1.03

**Table 11:** Dynamic viscosities of the biomass obtained from various sources.

Type of Raw Materials	Pure Glucose	Zharkent Glucose– Fructose Syrup	Burunday Glucose– Fructose Syrup
Shear viscosity, Pa∙s	2.3	3.2	4.4

## Data Availability

Not applicable.
